# Identification of a Functional Type VI Secretion System in *Campylobacter jejuni* Conferring Capsule Polysaccharide Sensitive Cytotoxicity

**DOI:** 10.1371/journal.ppat.1003393

**Published:** 2013-05-30

**Authors:** Nancy M. C. Bleumink-Pluym, Lieke B. van Alphen, Lieneke I. Bouwman, Marc M. S. M. Wösten, Jos P. M. van Putten

**Affiliations:** Department of Infectious Diseases & Immunology, Utrecht University, Utrecht, the Netherlands; The University of British Columbia, Canada

## Abstract

The pathogen *Campylobacter jejuni* is the principal cause of bacterial food-borne infections. The mechanism(s) that contribute to bacterial survival and disease are still poorly understood. In other bacterial species, type VI secretion systems (T6SS) are increasingly recognized to contribute to bacterial pathogenesis by toxic effects on host cells or competing bacterial species. Here we report the presence of a functional Type VI secretion system in *C. jejuni*. Proteome and genetic analyses revealed that *C. jejuni* strain 108 contains a 17-kb T6SS gene cluster consisting of 13 T6SS-conserved genes, including the T6SS hallmark genes *hcp* and *vgrG*. The cluster lacks an ortholog of the ClpV ATPase considered important for T6SS function. The sequence and organization of the *C. jejuni* T6SS genes resemble those of the T6SS located on the HHGI1 pathogenicity island of *Helicobacter hepaticus*. The *C. jejuni* T6SS is integrated into the earlier acquired *Campylobacter* integrated element CJIE3 and is present in about 10% of *C. jejuni* isolates including several isolates derived from patients with the rare clinical feature of *C. jejuni* bacteremia. Targeted mutagenesis of *C. jejuni* T6SS genes revealed T6SS-dependent secretion of the Hcp needle protein into the culture supernatant. Infection assays provided evidence that the *C. jejuni* T6SS confers contact-dependent cytotoxicity towards red blood cells but not macrophages. This trait was observed only in a capsule-deficient bacterial phenotype. The unique *C. jejuni* T6SS phenotype of capsule-sensitive contact-mediated hemolysis represents a novel evolutionary pathway of T6SS in bacteria and expands the repertoire of virulence properties associated with T6SS.

## Author Summary

Bacteria contain a number of secretion systems to export macromolecules to the environment. The bacterial type VI secretion system (T6SS) forms a needle-like structure that delivers toxic effector molecules to neighboring eukaryotic and/or prokaryotic cells. Here we report that the important human pathogen *Campylobacter jejuni* contains a functional T6SS gene cluster. The cluster comprises 13 conserved T6SS genes including genes encoding the typical T6SS Hcp and VgrG proteins. The gene cluster is part of a larger DNA element and is present in about 10% of *C. jejuni* strains including several blood isolates. The identified *C. jejuni* T6SS has unique properties compared to similar systems in other bacterial species. *C. jejuni* T6SS lacks the ClpV ATPase that supposedly energizes part of T6SS function in other species, causes contact-dependent lysis of red blood cells, and requires downregulation of the *C. jejuni* capsule polysaccharide to be effective. The unique cytotoxic properties of *C. jejuni* T6SS, the effect of the capsule on T6SS function, and the possible association with systemic *C. jejuni* infection broaden the scope of the existing bacterial T6SS phenotypes and point to a different evolution of *C. jejuni* T6SS compared to other bacterial species.

## Introduction

Gram-negative bacteria have evolved at least six types of protein secretion systems (type I–VI) to export proteins to the periplasmic space or the environment [1]. Several secretion systems are composed of needle-like structures that span the bacterial cell wall and protrude from the cell surface. These nanomachines include the classical type III and type IV secretion apparatus involved the injection of bacterial proteins into eukaryotic cells. One more recently discovered bacterial needle structure is the type VI secretion system (T6SS) as originally described for *Vibrio cholerae* and *Pseudomonas aeruginosa* [2], [3]. Today whole genome analyses have predicted T6SS gene clusters to be present in more than 100 Gram-negative bacterial species. These gene clusters often have of a variable composition but typically contain at least 13 core genes that encode the basic elements of the injection apparatus [4]–[6].

Structurally the T6SS consists of a membrane-associated assembly platform and a needle structure that transports effector molecules into neighboring bacteria or eukaryotic cells. A number of the T6SS core proteins show similarity to elements of tailed bacteriophages. Examples are the baseplate gp-25-like protein VCA109, the tail sheath-like proteins TssB and TssC (VipA/VipB), the tail subunit-like hemolysin co-regulated protein (Hcp) that polymerizes into the T6SS needle structure, and the valine-glycine repeat protein (VgrG) that forms the spike of the nanotube [4]–[6]. The structural similarity with bacteriophage proteins has led to the hypothesis that T6SS resemble an inverted bacteriophage tail structure that is exposed at the surface of the bacterial cell wall [7], [8]. Recently, contraction and extension of the VipA/B tubular sheath of the T6SS of *V. cholerae* have been visualized *in vivo*, supporting the model that the T6SS sheath is a dynamic contractile structure that projects the T6SS spike into the target cell analogous to bacteriophage entry [9], [10]. Disassembly of the contracted sheath requires the T6SS ClpV ATPase [9], [11], [12]. Another group of T6SS building blocks (TssM-L) seems related to proteins of the type IV secretion system (i.e. IcmF and IcmH/DotU) [13], [14]. These proteins may be involved in the recruitment of Hcp to the T6SS inner membrane assembly platform [15].

The hallmark of a functional T6SS is the presence of Hcp and VgrG in the culture supernatant [3], [16]–[18]. Both proteins may exert effector functions on eukaryotic cells [16], [19]–[21]. For the VgrG protein this function is often associated with the presence of an additional C-terminal effector module. Once in contact with eukaryotic cells, the extended C-terminal domain induces cross-linking or ADP-ribosylation of actin in eukaryotic cells, promoting intestinal inflammation and host cell toxicity [16], [19], [21]. Other identified T6SS effector molecules include the VasX protein secreted by *V. cholerae* that binds membrane lipids [22] and toxic proteins that target prokaryotes to provide a competitive advantage against other microorganisms occupying the same niche. Examples are the Tse2 toxin and the Tse1 and Tse3 proteins with peptidoglycan hydrolyzing activity in *Pseudomonas aeruginosa* [23]–[25]. These toxins may be representatives of a widespread T6SS effector superfamily with antibacterial properties [26].

*Campylobacter jejuni* is one of the principal bacterial food-borne pathogens causing millions of cases of gastroenteritis worldwide. Yet, the pathogenesis of *C. jejuni* infections is still poorly understood and a limited number of potential virulence determinants have been identified [27]. In the present study we report the identification of a functional T6SS in *C. jejuni*. The T6SS gene cluster is part of an integration element present in the genomes of a subset of *C. jejuni* strains. The system shows several unique traits compared to other bacterial T6SS including contact-dependent lysis of red blood cells and capsule expression-sensitive T6SS function.

## Results

### Identification of a *C. jejuni* Hcp ortholog

**Figure 1:**
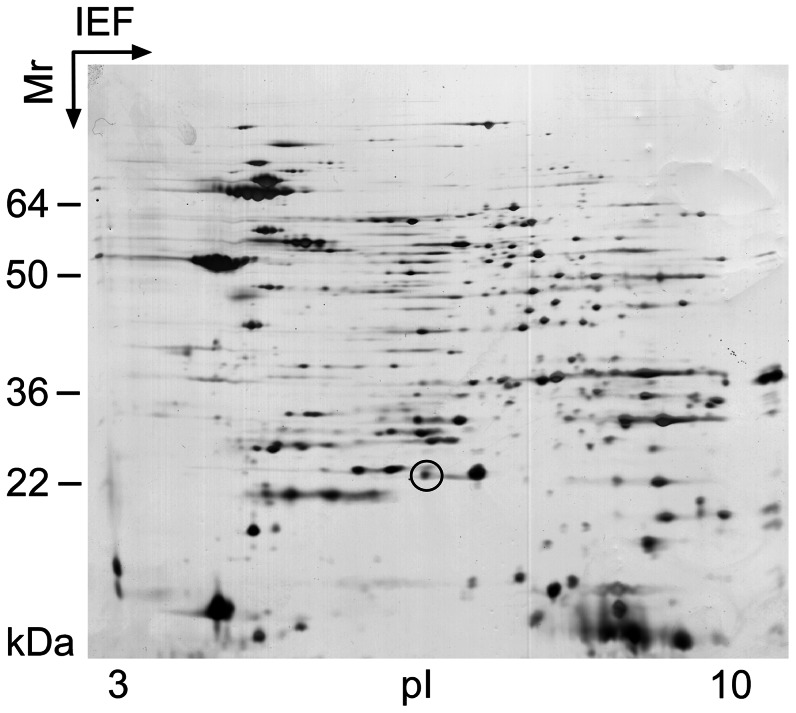
Two-dimensional SDS-PAGE of isolated whole bacterial lysate derived from *C. jejuni* strain 108. Proteins were visualized with silver. The encircled protein spot was picked and identified by mass spectrometry as a putative *C. jejuni* Hcp protein.

Proteome analysis of whole bacterial lysates of *C. jejuni* strain 108 using two-dimensional gel electrophoresis and liquid chromatography mass-spectrometry (LC-MS) revealed a ∼20 kDa protein that contained 4 peptide sequences most similar to a *Campyobacter coli* protein annotated in the NCBI database either as hypothetical protein or as putative hemolysin co-regulated protein (Hcp) (Fig. 1). We amplified the putative *C. jejuni hcp* gene from strain 108 by PCR with primers designed on the basis of the *C. coli hcp* sequence. Cloning and sequence analysis of the PCR product indicated that the *C. jejuni* gene encodes one open reading frame of 171 amino acids and contains the DUF796 domain which is conserved among Hcp proteins. The *C. jejuni* Hcp shows 76% similarity to the *V. cholerae* Hcp protein [28] and 69% similarity to the well characterized Hcp protein of *Pseudomonas aeruginosa* [2], [29].

### *C. jejuni* strain 108 contains a T6SS gene cluster

**Figure 2:**
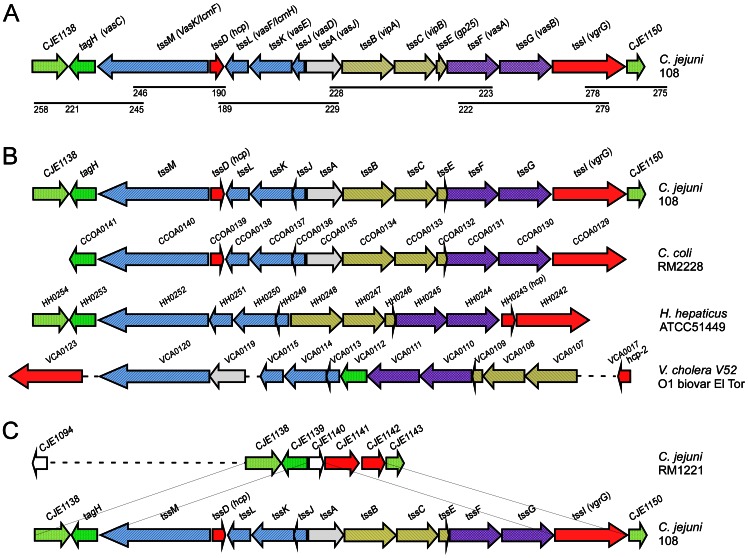
Gene organization of the T6SS clusters in *C. jejuni* strain 108 and related species. (A) The *C. jejuni* genes are designated according to the proposed nomenclature [5], [29] as *ttsA-M*; alternative gene names in *V. cholerae* are given in brackets for comparison. Functionally related group of genes are indicated in the same color. The lines and numbers below the colored genes indicate the PCR products and primers used for cloning and sequencing by primer walking. The same PCR primer sets were used to determine the gene organization in other Hcp-positive *C. jejuni* strains. (B) Comparison of the T6SS gene cluster organizations in *C. jejuni* strain 108, *C. coli* strain RM2228, *H. hepaticus* strain ATCC51449 and *V. cholerae* strain V52. Functionally related group of genes are indicated in the same color. (C) Diagram indicating the site of insertion of the *C. jejuni* 108 T6SS gene cluster relative to the CJIE3 element of *C. jejuni* RM1221.

In search for evidence of the presence of a complete T6SS gene cluster in *C. jejuni* strain 108, we determined the flanking regions of the *hcp* gene by primer walking. This strategy yielded a putative *C. jejuni* T6SS gene cluster of ∼17 kilobases consisting of 13 open reading frames with tight intergenic spacing (less than 30 bp) (Fig. 2A). The genes seemed organized in several groups based on gene orientation and were designated as *C. jejuni tssA-M* following the proposed nomenclature for T6SS components [5], [30]. The organization of the T6SS genes of *C. jejuni* strain 108 resembled but was not identical to the organization of the cluster in *C. coli* and *Helicobacter hepaticus* (Fig. 2B).

Closer inspection revealed that in the first group of genes the *C. jejuni hcp* gene (*tssD*) of strain 108 is located in reverse orientation between the *tssJ-M* genes (Fig. 2A). The products of these genes show 46–55% similarity with TssJ-TssM proteins which in several other species form essential components of the membrane platform of the T6SS secretion apparatus [13], [31]. *C. jejuni* TssJ (17 kDa) has a putative lipoprotein signal peptidase cleavage site (LFFCA/CSSVV) and a serine residue at position +2 which may sort the protein to the outer membrane [13], [32]. *C. jejuni* TssK (53 kDa) lacks an apparent signal sequence and resembles a conserved T6SS protein with unknown function. The putative *C. jejuni* TssL (30 kDa) and TssM (137 kDa) proteins are predicted to have transmembrane domains and coiled-coil structures. Both proteins share characteristics with the IcmH/DotU and IcmF proteins originally identified as non-essential components of a type IV secretion system (T4SS) that facilitate the translocation of bacterial effector proteins into eukaryotic target cells [33], [34]. These proteins are now considered conserved T6SS base plate components [13], [14], [35]. *C. jejuni* TssM contains a Walker A motif (GXXGXGKT/S) in its cytoplasmic N-terminal domain implicated in the ATP hydrolysis energizing the recruitment of Hcp to the TssL-TssM membrane complex [15]. The larger periplasmic part harbors an icmF domain that, in analogy to IcmH and IcmF, may interact with TssL and stabilize the secretion complex [13].

A second group of predicted T6SS components in *C. jejuni* strain 108 comprises the proteins TssB, TssC, TssE, TssF and TssG (Fig. 2B). TssB (18 kDa) and TssC (55 kDa) are orthologs of the T6SS proteins VipA and VipB that constitute the tubular sheath [10], [11]. *C. jejuni* TssE (15 kDa) has remote homology to the bacteriophage T4 baseplate protein gp25 and to the *V. cholerae* ortholog VCA0109 that is essential for Hcp secretion [11]. *C. jejuni* proteins TssF (66 kDa) and TssG (38 kDa), both predicted to be inner membrane proteins, are homologous to conserved T6SS components of unknown function, although the TssF ortholog in *Rhizobium leguminosarum* (ImpG) is involved in plant root infection [36]. The *C. jejuni* TssA (50 kDa) and TagH (35 kDa) proteins resemble T6SS hypothetical proteins. *C. jejuni* TagH appears to have a forkhead-associated domain (FHA) that may confer phosphoprotein-specific protein-protein interactions and thus may have a regulatory function. The *C. jejuni* T6SS gene cluster lacks a ClpV-ATPase ortholog implicated in the depolymerization and recycling of the T6SS tubular sheath proteins that may wrap the Hcp inner tube structure [9]–[12].

The protein encoded by the *tssI* gene located at the C-terminal end of the *C. jejuni* T6SS gene cluster shows similarity with the Rearrangement hot spot (Rhs) element of the VgrG protein family and with the bacteriophage T4 tail spike protein [7]. VgrG proteins form the T6SS needle tip and can puncture and translocate across eukaryotic membranes [16]. The VgrG-like protein of *C. jejuni* strain 108 lacks the extended biological effector domain that is often associated with modulation of eukaryotic cell function [16], [37]. The major characteristics of the T6SS gene cluster of *C. jejuni* strain 108 and its most related orthologs in several other species are summarized in Table 1.

**Table 1:** Characteristics of *C. jejuni* T6SS components and their most related orthologs in several other species.

*C. jejuni* strain 108			*V. cholerae* O1 biovar eltor	*H. hepaticus* 51449	*C. jejuni* RM1221	*C. coli* RM1228
Name	Size (aa)	COG	Ortholog (accession number)	Ortholog (accession number)	Ortholog (accession number)	Ortholog (accession number)
TssI (vgrG)	841	3501	vgrG VCA0123 (NP_232524)	HH0291 (AAP76839)	CJE1142 (AAW35468) CJE1141 (AAW35467)	CCOA0129 (EAL55897)
TssG	322	3520	vasB VCA0111 (AAF96025)	HH0244 (AAP76841)		CCOA0130 (EAL55898)
TssF	573	3519	vasA VCA0110 (AAF96024)	HH0245 (AAP76842)		CCOA0131 (EAL55899)
TssE (gp25)	130	3518	gp25 VCA0109 (NP_232510)	HH0246 (AAP76843)		CCOA0132 (EAL55900)
TssC (vipB)	484	3517	vipB VCA0108 (NP_232509)	HH0247 (AAP76844)		CCOA0133 (EAL55901)
TssB (vipA)	161	3516	vipA VCA0107 (NP_232508)	HH0248 (AAP76845)		CCOA0134 (EAL55902)
TssA	415	3515	vasJ VCA0119 (AAF96033)			CCOA0135 (EAL55903)
TssJ	148	3521		HH0249 (AAP76846)		CCOA0136 (EAL55904)
TssK	465	3522	vasE VCA0114 (AAF96028)	HH0250 (AAP76847)		CCOA0137 (EAL55905)
TssL (IcmH)	257	3455	vasF VCA0115 (AAF96029)	HH0251 (AAP76848)		CCOA0138 (EAL55906)
TssD (hcp)	171	3157	hcp VCA0017 (NP_232418)	HH0243 (AAP00840)		CCOA0139 (EAL55907)
TssM (IcmF)	1176	3523	vasK VCA0120 (AAF96034)	HH0252 (AAP76849)		CCOA0140 (EAL55908)
TagH	299	3456	vasC VCA0112 (AAF96026)	HH0253 (AAP76850)	CJE1139 (AAW35466)	CCOA0141 (EAL55909)

### The T6SS cluster is located on *C. jejuni* integrative element 3

T6SS gene clusters are usually located on pathogenicity islands or chromosomal regions that show a bias towards bacterial survival or virulence [5]. The G+C content of the *C. jejuni* T6SS is 26.5%, compared to about 30% for the *C. jejuni* genome. In *C. jejuni* strain 108 the T6SS cluster is flanked at the amino- and carboxyterminal ends by orthologs (98% similarity at the amino acid level) of respectively CJE1139 and CJE1141/CJ1142 of strain RM1221. These genes are located on CJIE3, an integrated element present in the genome of several *C. jejuni* strains including RM1221 [38]. The CJIE3 element of strain RM1221 lacks the T6SS gene cluster but contains CJE1141 and CJE1142 that have Rhs elements. Rhs elements can mediate chromosomal rearrangement or acquisition of new genetic information [39]. A fused homolog of these 2 genes forms the *tssL* (VgrG) gene at the carboxyterminal end of T6SS of *C. jejuni* strain 108. This gene organization strongly suggests that the T6SS of *C. jejuni* 108 is localized within CJIE3 and has inserted between the genes CJE1139 and CJE1141/CJE1142 of strain RM1221. Analysis of *C. jejuni* strain 108 for the presence of CJIE3 by PCR using a specific primerset [38] confirmed the presence of this element in strain 108. Fig. 2C shows a schematic representation of the insertion of the T6SS locus of *C. jejuni* strain 108 between genes CJE1139 and CJE1141/CJE1142 in the CJIE3 element of *C. jejuni* strain RM1221.

### Prevalence of *C. jejuni* T6SS

**Figure 3:**
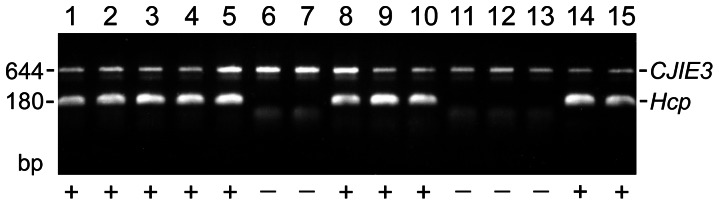
PCR detection of CJIE3 and Hcp in different *C. jejuni* and *C. coli* strains. Only CJIE3-positive strains are shown. *C. jejuni* strains used are 1 - 108, 2 - 202606, 3 - 209071, 4 - 205223, 5 - NCTC12502 (P3), 6 - RM1221, 7 - C09165, 8 - C019168, 9 - C626, 10 - C631, 11 -C10, 12 - 117, 13 - C356. *C. coli* strains used are: 14 - Han35, 15 - Han 153. Hcp-positive strains are marked with (+). The size of the PCR products is indicated in base pairs (bp). CJIE3 and *hcp* were not detected by PCR in the following *C. jejuni* strains: NCTC11168, NCTC81116, ATCC 33291, ATCC 49301, BAA527, BAA529, C013199, C011338, C017289, C011672, C011300, C013500, C012599, C012446, 5003, D3468, D3141, CCUG10950, D3226, 233.95, 308.95, 21.97, 386.96, 260.94, 41239B, 07479, 127955, 850312, 40707L, GB1, GB5, GB11, GB18, GB23, GB26, GB27, E98623, 480, 209071, 201191, 205224, 207251, 207252, 206470, 206710, 105713, 146719, 209755, 100756, 132960, 210388, 11271, 11279, 81176, A3004, C9, C12, C608, C618, C621, C627; and the *C. coli* strains: UA417, Han36, 2371, K1102/03, H1.

The localization of *C. jejuni* T6SS on genetic element CJIE3 in strain 108 and the variable presence of T6SS genes in other *C. jejuni* genomes led us to determine the T6SS prevalence in *C. jejuni*. To this end, we analyzed 80 *Campylobacter* strains for the presence of the *hcp* gene using PCR. Both human and animal isolates from different regions of the world were analyzed (Table S1). PCR products were obtained for eight *C. jejuni* and two *C. coli* strains (Fig. 3). Notably, four of T6SS-positive strains were isolates derived from patients with *C. jejuni* bacteremia which is a rare event that occurs in <0.2% of intestinal *C. jejuni* infections [40].

In order to investigate whether the *C. jejuni* strains containing the *hcp* gene also harbored the integrative element CJIE3, DNA from all 80 *Campylobacter* strains was analyzed by PCR using CJIE3-specific primers [37]. All *hcp* positive strains scored positive for CJIE3. However, we also identified several CJIE3-positive strains that lacked the *hcp* gene, like strain RM1221 (Fig. 3).

The organization and genomic integration of the T6SS cluster in the *hcp* positive *C. jejuni* strains was further characterized with primers used for the analysis of the T6SS gene cluster in strain 108. This confirmed that the complete T6SS locus was present in all the Hcp-positive *C. jejuni* strains. The T6SS clusters were flanked at the aminoterminal end by CJE1138 orthologs in 6 out of 8 *C. jejuni* strains and 1 of 2 *C. coli* strains. In all strains the carboxyterminal flanking region of T6SS was different than in strain 108 as no PCR products were obtained. Therefore we assume that the complete T6S locus has been acquired by *C. jejuni* in one step, while integration occurred at different positions of the earlier integrated element CJIE3.

### Functional characterization of the *C. jejuni* T6SS

**Figure 4:**
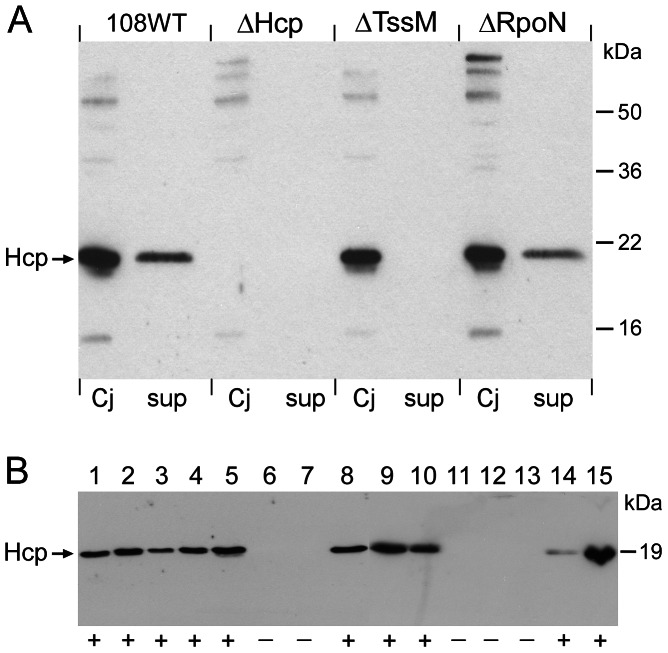
Western blot demonstrating the presence of Hcp protein. (A) Cellular (Cj) and culture supernatant (sup) fractions of *C. jejuni* strain 108 and its Hcp-, TssM- and RpoN-negative derivatives were separated by SDS-PAGE, blotted, and incubated with Hcp-specific antisera. Molecular mass markers are indicated in kilodalton (kDa). (B) Western blot of culture supernatants of the CJIE3-positive *C. jejuni* strains tested in Fig. 3, demonstrating secretion of Hcp by all T6SS-positive strains. Hcp-secreting strains are marked with (+). The apparent mass of the Hcp protein is indicated in kilodalton (kDa). The strains loaded in lanes 1 to 15 are listed in the legend of Fig. 3.

Evidence that the *C. jejuni* T6SS is functional was sought by analysis of Hcp secretion. Hereto the *hcp* gene was expressed in *Escherichia coli* SE1. A 6×His-tag was fused to the carboxy-terminal end of the protein for purification purposes. Rabbits were immunized with the purified recombinant protein to generate Hcp-specific antibodies. SDS-PAGE and Western blotting confirmed specific reactivity of the antiserum with Hcp (Fig. 4A). Immunoblotting of *C. jejuni* whole cell lysates and (non-concentrated) culture supernatants using the Hcp-specific antiserum demonstrated Hcp in both fractions (Fig. 4A). Secreted Hcp and cellular Hcp showed a similar apparent molecular mass, suggesting that no additional processing of the protein occurs during secretion. Inactivation of *hcp* by allelic replacement with a defective copy of the gene yielding strain 108ΔHcp, resulted in loss of immunoreactivity (Fig. 4A).

To gain evidence that Hcp secretion was conferred by the putative T6SS machinery, the *C. jejuni tssM* gene was inactivated keeping in mind that its ortholog in other species (e.g. VasK in *V. cholerae*, IcmF in *R. leguminosarum*) is required for T6SS-dependent Hcp secretion [3]. The mutant was constructed by replacement of the gene with a disrupted copy containing a chloramphenicol resistance cassette, yielding *C. jejuni* 108ΔTssM. The disrupted gene had inserted in the same orientation as the parent gene as verified by PCR. Western blot analysis of the TssM mutant demonstrated the presence of Hcp in whole bacterial lysates but not in the culture supernatant (Fig. 4A). These results strongly suggest that *C. jejuni* Hcp is secreted in a T6SS-dependent fashion. Immunoblots of culture supernatants of other *hcp*-positive *C. jejuni* strains demonstrated Hcp secretion for all of the tested strains (Fig. 4B).

The presence of large quantities of *C. jejuni* Hcp in the culture medium suggested constitutive expression of the T6SS genes under standard bacterial growth conditions. In *V. cholerae* and several other bacterial species, Hcp production requires the alternative transcription factor sigma-54 encoded by the *rpoN* gene and an enhancer binding protein (e.g. VasH) [3], [6], [41]. Although an ortholog of *vasH* appears absent from the T6SS locus of *C. jejuni* 108, we tested the effect of genetic inactivation of *C. jejuni rpoN* on Hcp expression. This mutant has a defect in flagella assembly and displays a motility deficient phenotype [42]. Immunoblotting of cellular and supernatant fractions of strain 108ΔRpoN revealed unaltered high levels of Hcp for the mutant and parent strain (Fig. 4A). These results suggest that RpoN does not regulate Hcp secretion in *C. jejuni*.

### *C. jejuni* T6SS causes cytotoxicity toward red blood cells

In search for a biological function of the *C. jejuni* T6SS we first tested the potential competitive advantage of *C. jejuni* strain 108 towards other microorganisms. In several bacterial species the presence of T6SS facilitates survival in mixed populations, often through the production of antibacterial toxins that are injected into neighboring bacteria [24], [26], [43], [44]. In our hands, co-culture of *C. jejuni* strain 108 with *E. coli* DH5α either in broth or on agar plates for up to 5 days did not reveal a selective growth advantage for the T6SS expressing strain. Similar results were obtained when *C. jejuni* strain 108 was incubated with the TS6SS-negative *C. jejuni* strain 81116ΔCPS which has similar growth requirements and growth rate as strain 108.

**Figure 5:**
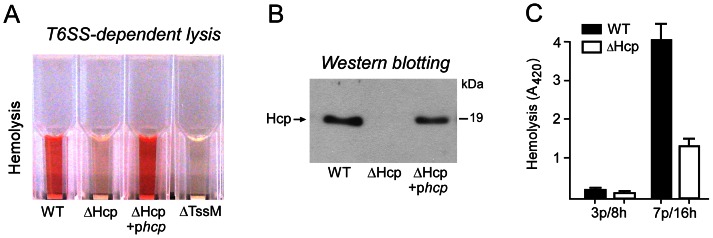
Hemolysis assay depicting the T6SS-mediated hemolytic activity of C. **jejuni.** (A) *C. jejuni* strain 108, its hcp-negative derivative 108ΔHcp, the complemented mutant strain 108ΔHcp/p*hcp*, and the parent strain carrying p*hcp* grown for 7-days on saponin plates and then for 16 hours in HI broth (7 p-16 h) were incubated (6 h) with red blood cells before hemolysis was determined. (B) Western blot confirming the successful restoration of Hcp secretion in 108ΔHcp after introduction of the complementation plasmid p*hcp*. (C) Effect of the age of *C. jejuni* cultures in T6SS-induced hemolysis. Strain 108 and 108ΔHcp grown either on saponin plates for 3 days and then in HI broth for 8 h (3 p/8 h), or on saponin plates for 7 days and then in HI broth for 16 h (7 p/16 h) were incubated with red blood cells for 6 h. Then hemolysis was determined by measuring absorbance at 420 nm. Values are the mean ± SEM of at least three experiments.

Although *C. jejuni* T6SS appeared to lack the extended VgrG protein often associated with cytotoxicity toward host cells, we next tested the effects of *C. jejuni* strains 108 and 108ΔHcp on eukaryotic cells including Caco-2 intestinal epithelial cells and red blood cells. Confocal laser microscopy on Caco-2 cells infected with strain 108 demonstrated that internalized *C. jejuni* remained in a CD63-positive endolysosomal compartment for up to 24 h [45], suggesting that T6SS apparatus did not cause lysis and bacterial escape from the intracellular vacuole. The effect of the T6SS on erythrocytes was assessed by measurement of hemolytic activity after 6 h of incubation of the red blood cells with *C. jejuni*. This showed that *C. jejuni* 108 caused strong hemolysis compared to mutant strain 108ΔHcp (Fig. 5A). Complementation of the *hcp* mutant by the introduction of plasmid pMA1-*hcp* carrying an intact copy of the *hcp* gene restored the strong T6SS-associated cytotoxicity (Fig. 5**A**). We also tested the *tssM* mutant for hemolytic activity. This mutant failed to induce Hcp-induced hemolysis consistent with the observed essential function of the TssM protein in Hcp secretion (Fig. 5A). Western blotting confirmed that the complementation of the *hcp* mutant and inactivation of *tssM* resulted the expected changes in Hcp secretion (Fig. 5B). Isolated culture supernatant of strain 108 lacked hemolytic activity, suggesting that the T6SS phenotype involved contact-dependent hemolysis. It is important to note that the T6SS-induced hemolysis was observed for *C. jejuni* grown on agar plates for 7 days and then in HI broth for 16 h (7 p/16 h)(Fig. 5C). *C. jejuni* grown on agar plates for 3 days followed by growth in HI broth for 8 h (3 p/8 h) failed to consistently induce substantial hemolysis (Fig. 5C).

### Capsule impairs T6SS function

**Figure 6:**
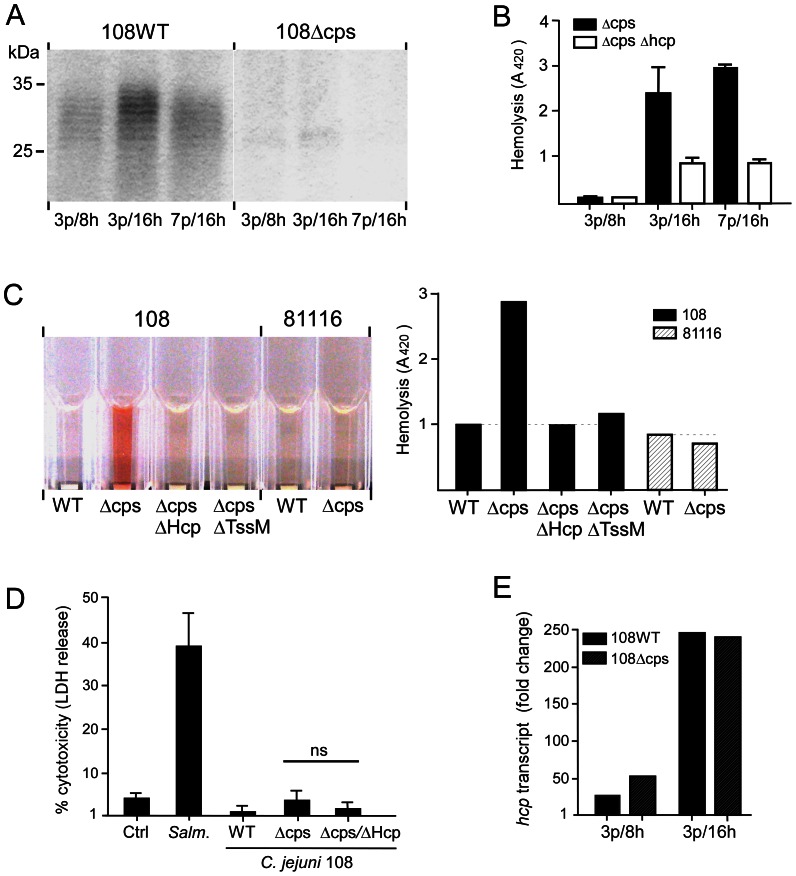
Effect of capsule expression on T6SS-mediated hemolysis. (A) SDS-PAGE of *C. jejuni* strain 108 and its capsule-negative mutant 108ΔCPS stained for the presence of capsule polysaccharide with Alcian blue. Capsule expression was compared for equal amounts of *C. jejuni* grown on saponin plates for three (3 p) or seven (7 p) days and then in HI broth for the indicated times (8 h or 16 h). Molecular mass markers are indicated in kilodalton (kDa). (B) T6SS-mediated hemolysis (6 h) for *C. jejuni* strain 108 and 108ΔHcp in a capsule-negative background (ΔCPS). Hemolysis was determined by measuring absorbance at 420 nm. Values are the mean ± SEM of at least three experiments. (C) Hemolysis assay depicting the hemolytic activity (6 h) of *C. jejuni* strain 108 (WT), the capsule mutant 108ΔCPS, the double mutant 108ΔCPS/ΔHcp, and the Hcp secretion-defective strain 108ΔTssm, and of the T6SS-negative strain 81116 (WT) and its capsule mutant 81116ΔCPS. Strains used were grown for 3 days on saponin plates and then for 16 h in HI broth (3 p/16 h). The hemolysis results depicted in the left panel were quantified by measuring absorbance at 420 nm (right panel). (D) Effect of T6SS on J774A.1 macrophages. Macrophages were incubated with *C. jejuni* strain 108, 108ΔHcp, 108ΔCPS, 108ΔCPS/ΔHcp or S. Typhimurium SL1344. After 10 h of incubation, total cellular and released lactate dehydrogenase (LDH) were measured as indicator of cytotoxicity. Values are the mean ± SEM of at three independent experiments performed in triplicates. (E) Real-time RT-PCR results showing *hcp* transcript levels for *C. jejuni* strain 108 and 108ΔCPS at different age (3 p/8 h and 3 p/16 h). Note the strong relative increase in *hcp* transcript level in late exponential growth phase. Data are representative of three independent experiments with two independent preparations of RNA.

In an attempt to understand the apparent bacterial growth-related variation in T6SS phenotype, we hypothesized that perhaps the *C. jejuni* surface capsule polysaccharide (CPS) interfered with the contact-dependent hemolysis. To test this hypothesis we first analyzed the CPS of *C. jejuni* 108 at different age of culture using Alcian blue staining. This revealed variable intensity of capsule staining with highest levels of capsule expression for *C. jejuni* grown on agar plates for 3 days and then in HI broth for 16 h (3 p/16 h) (Fig. 6A). At earlier (3 p/8 h) and later (7 p/16 h) time points, CPS levels were much lower (Fig. 6A). This seemed to exclude variable CPS expression as a cause of the observed variation in T6SS-mediated hemolysis at these time points (Fig. 5C).

To definitively exclude the capsule as an inhibitory factor of T6SS function, we tested the capsule-deficient strain *C. jejuni* 108ΔCPS for hemolytic activity. The mutant was constructed by allelic exchange of the *kpsM* gene with a disrupted copy of this gene. Unexpectedly, *C. jejuni* 108ΔCPS displayed strong cytotoxicity at all tested time points except during early exponential growth phase (3 d/8 h) (Fig. 6B). The cytotoxic effect of the CPS mutant was abolished after additional inactivation of *hcp* or *tssM* (Fig. 6C), indicating that the hemolysis required a functional T6SS. Genetic inactivation of *kspM* in *C. jejuni* 81116, which lacks the T6SS gene cluster, did not result in enhanced hemolysis (Fig. 6C). Together, these results demonstrate that *C. jejuni* T6SS confers a cytotoxic phenotype toward red blood cells but that this function requires downregulation of the polysaccharide capsule. Notably, similar experiments with strains 108, 108ΔCPS, 108ΔHcp and 108ΔCPS/ΔHcp and J774A.1 macrophages caused minimal cell damage (i.e. LDH release), irrespective the presence of a functional T6SS (Fig. 6D).

The issue that remained to be resolved was why the 3 d/8 h *C. jejuni* culture fails to show the T6SS phenotype even in the capsule-negative background. To address the point, we compared relative transcript levels of *hcp* in 3 p/8 h and 3 p/16 h cultures for *C. jejuni* strains 108 and 108ΔCPS. Real-time PCR analysis showed 5–10 fold more *hcp* transcript for the 3 p/16 cultures compared to 3 p/8 h bacteria both for the parent and *cps* mutant strain (Fig. 6E). These results indicate that *C. jejuni hcp* mRNA levels vary between growth conditions and suggest that in the early logarithmic phase *C. jejuni* Hcp levels may be insufficiently expressed to induce the hemolytic phenotype.

## Discussion

Type VI secretion systems are bacterial nano-injection machines that transport macromolecules into neighboring prokaryotic or eukaryotic cells. The toxic effector molecules serve to outcompete other bacterial species [24], [43] or to alter host cells during pathogenesis [36], [46; for review see] [47]. Here we report the existence and function of a T6SS pathogenicity island in the principal bacterial food-borne pathogen *C. jejuni*. The T6SS gene cluster is present in about 10% of the *C. jejuni* isolates and has inserted into *C. jejuni* integrated element 3 (CJIE3). The function is evidenced by the secretion of the hallmark hemolysin co-regulated protein Hcp. *C. jejuni* T6SS is special among the T6SS family because it confers contact-dependent cytotoxicity toward red blood cells and because its function requires down-regulation of the polysaccharide capsule. The capsule controlled cytotoxicity of *C. jejuni* T6SS adds a new element to the growing repertoire of T6SS regulation mechanisms and phenotypes.

The T6SS cluster of *C. jejuni* consists of 13 genes that most resemble the T6SS genes of *C. coli* and *H. hepaticus* both with regard to gene organization and content (Fig. 2). In addition to the hallmark Hcp and VgrG-like proteins, predicted *C. jejuni* T6SS proteins include the TssJ-M proteins encoding base plate components of the secretion apparatus [13], and TsB (VipA), TssC (VipB) and TssE (gp25 protein) that form structures that resemble the evolutionary related bacteriophage tail sheath and baseplate proteins [4], [5]. The *C. jejuni* T6SS locus lacks the frequently found TssH gene (COG0542) encoding a ClpV ATPase implicated in the recycling of the TssB/TssC tubular sheath [9], [11]. Although important for sheath contraction and recycling, ClpV is not essential for T6SS function in *V. cholerae* [48]. Alternatively, it is possible that a related member of the ClpB family of ATPases encoded from elsewhere on the *C. jejuni* genome partakes in T6SS function, although analysis of the (incomplete) genome of strain 108 has thus far failed to detect a ClpV homologue in strain 108 (unpublished results).

The *C. jejuni* T6SS genes are only present in isolates that carry the integrative element CJIE3 (integrated into the 3′ end of an arginyl-tRNA). This element is likely plasmid derived and appears to consist of modular regions of unknown function that differ between CJIE3-positive strains [38]. The prevalence of CJIE3 among our 80 tested *C. jejuni* isolates and those used in the Parker study, is approximately 18%. Yet, only approximately 10% of the isolates contained the T6SS locus. Based on the difference in T6SS flanking regions in our isolates, we assume that the T6SS genes have been acquired *en bloc* and inserted at different positions into the previously acquired CJIE3.

The function of *C. jejuni* T6SS as a secretion apparatus is evident from the accumulation of Hcp in the culture medium and the defective Hcp secretion (but intact production) in the constructed *tssM*-defective strain (Fig. 4A). It is noteworthy that Hcp secretion was apparent under most routine laboratory growth conditions. In other bacterial species T6SS function often appears in response to distinct environmental cues. Although the signals triggering the expression of T6SS genes are still unknown, environmental conditions like temperature, pH, iron or the presence of host cells may influence their induction [49]. Involved regulatory systems include the sensor kinase RetS in *Pseudomonas aeruginosa* [50] and the *Burkholderia mallei* VirAG two-component system and AraC-type activator BMAA1517 [51]. Expression of T6SS gene clusters in *Vibrio cholerae*, *Aeromonas hydrophyla* and *Pseudomonas syringae* is regulated by sigma-54 and cognate enhancer binding proteins (e.g. VasH) [48]. We show that sigma-54 is not required for *C. jejuni* T6SS protein secretion (Fig. 4A). The molecular basis of the relative poor Hcp expression in the early exponential growth phase remains to be determined. A plausible alternative explanation for the limited gene regulation of *C. jejuni* T6SS may be this pathogen has evolved the described alternative strategy of capsule-sensitive T6SS function.

Our results indicate that *C. jejuni* T6SS causes contact-dependent lysis of red blood cells. Bacterial competition assays with T6SS-negative *C. jejuni* and *E. coli* yielded no conclusive T6SS phenotype under the conditions employed. Furthermore, incubation of *C. jejuni* 108ΔCPS with J774A.1 macrophages did not result in cytotoxicity (Fig. 6D) and the presence of T6SS did not seem to enable the intracellular *C. jejuni* to escape from the endolysosomal compartment [45]. Several lines of evidence indicate that the observed hemolysis was caused by T6SS activity: (i) Hemolysis was strongly reduced in the *hcp* and *tssM* mutants, (ii) complementation of the *hcp* mutant with an intact copy of the gene restored the phenotype, (iii) hemolysis was minimal for strain 81116 that lacks the T6SS gene cluster. The low level of hemolysis measured after prolonged incubation with the mutant strains (Fig. 6) may be attributed to the presence of membrane bound hemolysins, such as phospholipase A [52]. The strong T6SS-mediated hemolytic activity may benefit *C. jejuni* by increasing the availability of nutrient sources such as iron and nicotinamide adenine dinucleotide (NAD) that are abundantly present in erythrocytes.

A key feature in establishing the *C. jejuni* T6SS phenotype was the explanation of the initially highly variable results by changes in the level of capsule expression. The use of constructed capsule- and Hcp-negative mutant strains unequivocally demonstrate *C. jejuni* polysaccharide capsule as a key determinant controlling T6SS-induced hemolysis. To our knowledge capsule expression has not previously been implicated as a factor influencing the activity of T6SS. We propose that the polysaccharide capsule inhibits T6SS function by acting as a steric barrier that prevents the T6SS needle structure to puncture the host cells. In addition, needle length may be of importance as suggested by the poor hemolytic activity of capsule-negative strains at early growth phase when *C. jejuni hcp* transcript levels were much lower (Fig. 6). Thus, capsule thickness in conjunction with needle length may determine T6SS function in *C. jejuni*, reminiscent of the control of the function of the type III injectisome by needle length [53] and the extent of LPS glucosylation in *Shigella flexneri* [54]. The factors that regulate capsule expression in *C. jejuni* are not well defined [27], although recent studies indicate that downregulation of capsule does occur upon contact with host cells [55], [56]. In addition, at least in some *C. jejuni* strains capsule biosynthesis is subject to phase variation [57]. This event has not been demonstrated to occur in strain 108, but, when present, may result in a subset of the bacterial population with a functional T6SS phenotype. This scenario may explain the basal level of hemolysis observed for the wildtype *C. jejuni* strain 108.

An intriguing issue is as to why a subset of *C. jejuni* strains has acquired and maintains a seemingly genetically stable and functional T6SS pathogenicity island. Its relatively low prevalence (10%) and absence in many disease isolates indicate that the T6SS is not an essential virulence factor in intestinal infections but rather may be advantageous for the bacterium in a distinct niche. We did note that four out of the eight TSS6-positive *C. jejuni* isolates identified in this study were derived from human patients with *Campylobacter* bacteremia. This seems disproportionally high considering that *C. jejuni* bacteremia occurs in <0.2% of intestinal infections, often in immunocompromised patients [40]. We tested *C. jejuni* growth in human blood from healthy donors. This resulted resulted in rapid bacterial killing irrespective the presence of T6SS, indicating that in healthy donors bacterial killing mechanisms dominate over the possible growth advantage of T6SS-positive strains due to e.g. the release of nutrients from red blood cells. Similar assays in horse blood revealed no differences in bacterial growth/survival between strain 108 and the T6SS mutant (Fig. S1). The T6SS-positive *C. jejuni* blood isolates identified in the present study were all derived from patients with hypogammaglobulinemia, which may limit rapid bacterial killing. Yet, considering the low number of available blood isolates, we feel it too early to conclude that T6SS-mediated hemolysis contributes to the development of *C. jejuni* bacteremia.

The presence of a functional T6SS in *C. jejuni* has recently been confirmed in a parallel study using strain 43431 [58]. The T6SS gene cluster in this strain also lacks a ClpV homologue. Inactivation of T6SS function in this strain resulted in increased resistance to high concentrations of bile salts, possibly by preventing entry of bile salt through the opened secretion channel [58]. The mutant also showed approximately 50% reduced bacterial adhesion and invasion of host cells. Whether the observations with this strain also varied with the presence of the polysaccharide capsule was not investigated.

Overall, our study provides the first evidence that (i) ∼10% of *C. jejuni* isolates carry a complete T6SS gene cluster in the integrative element CJIE3, (ii) the T6SS system is functional, (iii) *C. jejuni* T6SS confers cytotoxicity toward red blood cells, and (iv) the T6SS phenotype requires down regulation of the polysaccharide capsule. To our knowledge, T6SS-mediated hemolysis and an effect of capsule on T6SS function has never been reported for other bacterial species. *C. jejuni* T6SS represents a novel direction in the evolution of T6SS, expands the existing repertoire of T6SS-mediated effects on eukaryotic cells, and may contribute to systemic *C. jejuni* infection.

## Materials and Methods

### Bacterial strains and growth conditions

The bacterial strains used in this study are listed in Supporting Information (Table S1). *Campylobacte*r was grown at 37°C under micro-aerobic conditions (5% O_2_, 10% CO_2_, 85% N_2_) either on Saponin agar plates containing agar base II medium (Oxoid Ltd., Basingstoke, UK) with 5% saponin lysed horse blood, or in 5 ml of Heart Infusion broth (HI) (Oxoid) in 25 cm^2^ tissue culture flasks at 160 rpm. *Escherichia coli* were grown in Luria-Bertani medium at 37°C. When appropriate, growth media were supplemented with ampicillin (100 µg/ml), kanamycin (20 µg/ml), or chloramphenicol (20 µg/ml).

### Genetic analysis of T6SS locus

The nucleotide sequence of the primers used for cloning and sequencing the T6SS locus are listed in Supporting Information (Table S2). DNA fragments encoding T6SS genes were PCR amplified by primer walking using 500 ng of isolated *C. jejuni* 108 genomic DNA as template, 2.5 U of Super Taq+ polymerase (HT Biotechnology Ltd. UK), 50 pmol of each primer, and 0.1 mM of dNTPs in a final volume of 50 µl of 10 mM of Tris-HCl, 1.5 mM of MgCl_2_ and 50 mM KCl. Standard PCR conditions were heating for 2 min at 95°C, followed by 35 cycles of 15 s at 95°C, 15 s at 52°C, and 5 min at 72°C in a BioRad iCycler. Amplified products were subjected to agarose gel electrophoresis, purified with the Qiaex II gel extraction kit (Qiagen), and cloned using pGEM-Teasy (Promega) or PJet (Fermentas) in *E. coli* DH5α. Primers T7 and Sp6 were used to determine the nucleotide sequences of the pGEM-Teasy inserts. The PJet1.2 forward and reverse primers were used to sequence the pJet1.2 inserts (Baseclear, Leiden, The Netherlands). Sequences were analyzed and aligned using the DNASTAR software package (Lasergene). The complete sequence of the T6SS gene cluster of *C. jejuni* strain 108 is deposited at GenBank (Accession number: JX436460).

### Construction of *C. jejuni* mutants

Primers used for construction of mutants are listed in Supporting Information Table S3. The *hcp* and *tssM* gene with their flanking sequences of strain 108 were amplified by PCR as described above. PCR products were cloned into pGEM-T easy. Inverse PCR on pGEM*hcp* was used to delete 166 bp of *hcp* and to introduce a *Bam*HI restriction site. Plasmid pGEM*tssM* was cut with *bgl*II. The chloramphenicol resistance gene (*Cm*) from pAV35 was ligated into the created *Bam*HI site in *hcp* and into the *Bgl*II site in *tssM*, yielding pGEM*hcp::cm* and pGEM*tssM::cm*, respectively. Knockout plasmids carrying the *Cm* gene in the same orientation as the *hcp* and *tssM* genes were used to transform *C. jejuni* 108 by electroporation. Mutants were selected on saponin agar plates containing 20 µg/ml of chloramphenicol. Disruption of the genes was verified by PCR.

For construction of *C. jejuni* 81116ΔCPS the *kpsM* gene with flanking sequences was amplified with primers listed in Table S3 and cloned into pGEM-T easy. A deletion of 300 bp was made using inverse PCR thereby creating a unique *Bgl*II restriction site. The 2,000 bp tetracycline resistance gene from pTetO was inserted into this *Bgl*II site, yielding pGEM-T easy *kpsM::tet*. Natural transformation was used to introduce the knockout plasmid into *C. jejuni* 81116. Mutants were selected on saponin agar plates containing 15 µg/ml of tetracycline. *Bgl*II digestion was used to replace the *tetO* gene in the pGEM-T easy *kpsM::tet* construct with the *Cm* gene from pAV35. The resulting plasmid pGEM*kpsM::cm* was introduced in *C. jejuni* 108 via electroporation, yielding strain 108ΔCPS. Chloramphenicol resistant transformants were selected and gene disruption was confirmed by PCR.

### Complementation of the *hcp* mutant

The *hcp* gene including its ribosomal binding site was PCR amplified from strain *C. jejuni* 108 using primers 276 and 277. After digestion with *Xho*I and *Xba*I, the resulting 531 bp fragment was cloned into the pMA1 vector behind the *C. jejuni metK* promoter [59], yielding pMA1*hcp*. The plasmid was introduced by electroporation into *C. jejuni* 108 wild type and the *hcp* mutant, and kanamycin resistant transformants were selected.

### Cloning, expression, and purification of recombinant Hcp

The *hcp* gene was PCR amplified from *C. jejuni* 108 genomic DNA with primers 210 and 211 and fused to a C-terminal 6× histidine tag using the *Xba*I–*Xho*I sites of pSCodon1 (Delphi genetics SA). Selection of pSCodon1*hcp* was performed in *E. coli* CYS21, while *E. coli* SE1 was used to express the Hcp protein (Staby Codon T7 manual, Delphi genetics SA). An overnight culture of *E. coli* pSCodon1*hcp* in Staby Switch auto-inducible medium (Eurogentec, Belgium) was used to isolate His-tagged Hcp from the soluble cytosolic fraction. Recombinant protein was purified under native conditions using Ni-NTA agarose and 250 mM of imidazole in the elution buffer as described in the manual (Qiagen). Eluted fractions were analyzed by SDS-PAGE. Hcp-positive fractions were pooled and dialyzed for 20 h against 3 liter of 50 mM of Tris-HCl buffer (pH 7.6) containing 200 mM of KCl, 10 mM of MgCl_2_, 0.1 mM of EDTA, 10% glycerol, and then against the same buffer containing 50% glycerol.

### Hcp antibody production

Two New Zealand White rabbits (SPF) were immunized four times (Days 0, 14, 28 and 56) with 100 µg of purified Hcp protein using the classical anti-protein protocol (Eurogentec, Belgium). Serum aliquots were collected (Days 0, 38, 66 and 87) and stored at −20°C. Dilutions of sera were tested by Western blot analysis for Hcp reactivity. All immunizations and handling of animals were performed by Eurogentec, Belgium.

### Western blot analysis

For protein detection, aliquots of bacterial cultures (16 h, HI broth) were pelleted (4,000× g, 15 min) and dissolved in the same volume of Laemmli electrophoresis solution containing 30 mM of Tris-HCl (pH 6.8), 4% SDS, 0.025% bromophenol blue and 20% glycerol. The bacterial supernatant was subjected to high speed centrifugation (18,500× g, 15 min) to remove supramolecular structures and mixed at a 3∶1 (v/v) ratio with 3×-concentrated Laemmli solution. After boiling (10 min), the equivalent of 10 µl of bacterial culture was loaded onto a 12% SDS-polyacrylamide gel. Proteins were transferred to PVDF membranes (Immobilon-P, Millipore). After blocking (5% skim milk powder (Elk, Campina), 0.1% Tween 20), Hcp was detected with rabbit anti-Hcp antiserum (1/500 in PBS, 2% skim milk, 0.1% Tween 20) in combination with horseradish peroxidase-conjugated goat anti-rabbit IgG (Santa Cruz Biotechnology) and Super signal west pico chemiluminescent substrate (Pierce).

### Two-dimensional gel electrophoresis and mass spectrometry

Two-dimensional gel electrophoresis was generously carried out by Dr. Bas van Balkom as previously described [60], except that precast immobilized nonlinear pH (pH 3 to 10) gradient strips (Amersham Biosciences) were used. In-gel tryptic digestion and mass spectrometry analysis were performed as described [60].

### Detection of polysaccharide capsule

For capsule detection, bacteria (2×10^9^) were suspended in 100 µl of Laemmli buffer. Next, 30 µl of 20 mg/ml proteinase K in water was added and the mixture was incubated at 55°C for 2 h. Capsular polysaccharides were separated on a 12% SDS-PAGE gel. Gels were washed twice in water and stained (1 h) with filtered 0.5% Acian Blue 8GX (Sigma) in 2% acetic acid/40% methanol. Gels were destained in 2% acetic acid/40% methanol until bands became visible [60]. SDS-PAGE and protein staining of non-digested aliquots of the samples confirmed equal loading of bacteria.

### Cytotoxicity assays

*C. jejuni* grown on saponin agar plates for 3 or 7 days were transferred to HI broth (OD_550_: 0.05) in 25 cm^2^ flask and shaken (160 rpm) for 8 h or 16 h at 37°C under microaerophillic conditions. Bacterial pellets from solid and broth media were suspended in PBS to OD_550_ of 1. One ml of this bacterial suspension was then mixed with 0.25 ml of a 5% (v/v) horse erythrocyte suspension (Biotrading) kept in PBS with 0.4 mM of CaCl_2_ in a 1.5 ml plastic tube with a perforated cap. After incubation (37°C, 6 h, microaerophilic conditions), the tubes were mixed and centrifuged (1,000× *g*, 5 min). The OD_420_ of the supernatants was then measured as indicator of the degree of hemolysis. Negative (with PBS without bacteria) and positive (with added water instead of bacterial suspension) controls were included in all assays. Cytotoxicity was scored as percentage of cell lysis of the positive control. Data are expressed as the mean ± SEM of at least three independent experiments.

Cytotoxicity toward macrophages was determined using J774A.1 macrophages (ATCC) grown for 24 h in a 24-well plate in DMEM+10% FCS at 37°C in a 10% CO_2_ atmosphere. Prior the use the medium was replaced by 1 ml of fresh DMEM (without FCS). *C. jejuni* strains 108, 108ΔHcp, 108ΔCPS, 108ΔCPS/ΔHcp and (as control) *Salmonella* Typhimurium strain SL1344 were grown in HI broth, collected by centrifugation (10 min, 3,000× *g*), and added to the macrophages at a bacteria to host cell ratio of 20. After 2 h of incubation (37°C, 5% CO_2_), the extracellular *Salmonella* were removed and 1 ml of DMEM+50 µg/ml gentamicin was added. The cells were incubated for an additional 10 h before total cellular LDH and LDH release from the cells was determined with the Cytotoxicity Detection Kit according to the protocol of the manufacturer (Roche). The supernatant or the lysed cells stained with the kit was analyzed on the FLUOstar omega (BMG Labtech) at 492 nM and 690 nM for wavelength correction. Cytotoxicity was determined by calculating the percentage of LDH released after background subtraction. *C. jejuni* had no effect on the LDH itself or the assay. Presented results are from three individual assays performed in triplicate. Data were analyzed using Graphpad Prism software.

### Survival of *C. jejuni* in blood

*C. jejuni* strain 108, 108ΔCPS and 108ΔCPS/ΔHcp grown in HI broth (16 h, 37°C) were collected by centrifugation (15 min, 4000× *g*) and suspended in 50 µl of HI broth. Bacteria (10^9^ CFU) were, added to 3 ml of heparinized human or defibrinated horse blood in 35 mm petri dishes and incubated at 37°C under micro-aerobic conditions. After 0, 6, 24 and 48 h, 10 µl aliquots were taken, serially diluted in PBS, and plated onto saponin agar plates. Bacterial survival was determined by counting the number of colony forming units after 48 h of incubation at 37°C under micro-aerobic conditions.

### Real-time RT-PCR

Total bacterial RNA was extracted from *C. jejuni* strain 108 and 108ΔCPS grown in 25 ml of HI Broth for 8 or 16 h at 37°C under micro-aerobic conditions, using the RNA-Bee kit (Tel-Test, Inc) according to the manufacturer's specifications. Isolated RNA was treated with 1 µg of DNase (Fermentas) per µg of RNA for 30 min at 37°C, after which the DNase was inactivated by heating at 65°C for 10 min in the presence of EDTA (2.5 mM final concentration). Real time RT-PCR analysis was performed using the Brilliant III Ultrafast SYBR Green QRT-PCR master mix (Agilent, Stratagene Inc). The PCR mixture (20 µl) contained 40 ng of DNase treated RNA, 20 pmol of the *Hcp* gene specific primers hcp-RT forw (5′-ACCCGATTTATATCTATTGCCAAT-3′) and hcp-RT rev (5′-GAAGGTTCCACACAAGGTTTGAT-3′), 10 µl of 2× SYBR green mix, 0.2 µl of 100 mM of DTT and 1 µl of RT/RNAse block. The reverse transcriptase cycle was 50°C for 10 min, followed by a PCR initial activation step of 95°C for 3 min. The mixtures were then amplified in 45 cycles of 95°C for 5 sec and 60°C for 10 sec in a Light Cycler 480 (Roche). Total *hcp* mRNA in each sample was normalized against the internal controls *gyrA* and *rpoA*. Three independent experiments with two independent preparations of RNA were analyzed by real-time RT-PCR.

## Supporting Information

Figure S1 **Growth of** ***C. jejuni*** **strain 108, 108ΔCPS and 108ΔCPS/ΔHcp in blood.**
(TIF)

Table S1 **Strains and plasmids used in this study.**
(DOC)

Table S2 **Primers used for cloning and sequencing of the T6SS locus of** ***C. jejuni*** **strain 108.**
(DOC)

Table S3 **Primers used for construction of mutants and expression vectors.**
(DOC)

## References

[1] Saier MH Jr (2006) Protein secretion and membrane insertion systems in gram-negative bacteria. J Membr Biol 214: 75–90. 17546510 10.1007/s00232-006-0049-7

[2] Mougous JD, Cuff ME, Raunser S, Shen A, Zhou M, et al (2006) A virulence locus of *Pseudomonas aeruginosa* encodes a protein secretion apparatus. Science 312: 1526–1530. 16763151 10.1126/science.1128393 PMC2800167

[3] Pukatzki S, Ma AT, Sturtevant D, Krastins B, Sarracino D, et al (2006) Identification of a conserved bacterial protein secretion system in *Vibrio cholerae* using the *Dictyostelium* host model system. Proc Natl Acad Sci U S A 103: 1528–1533. 16432199 10.1073/pnas.0510322103 PMC1345711

[4] Bingle LE, Bailey CM, Pallen MJ (2008) Type VI secretion: a beginner's guide. Curr Opin Microbiol 11: 3–8. 18289922 10.1016/j.mib.2008.01.006

[5] Cascales E (2008) The type VI secretion toolkit. EMBO Rep 9: 735–741. 18617888 10.1038/embor.2008.131 PMC2515208

[6] Silverman JM, Brunet YR, Cascales E, Mougous JD (2012) Structure and regulation of the type VI secretion system. Annu Rev Microbiol 66: 453–472. 22746332 10.1146/annurev-micro-121809-151619 PMC3595004

[7] Kanamaru S (2009) Structural similarity of tailed phages and pathogenic bacterial secretion systems. Proc Natl Acad Sci U S A 106: 4067–4068. 19276114 10.1073/pnas.0901205106 PMC2657389

[8] Leiman PG, Basler M, Ramagopal UA, Bonanno JB, Sauder JM, et al (2009) Type VI secretion apparatus and phage tail-associated protein complexes share a common evolutionary origin. Proc Natl Acad Sci U S A 106: 4154–4159. 19251641 10.1073/pnas.0813360106 PMC2657435

[9] Basler M, Mekalanos JJ (2012) Type 6 secretion dynamics within and between bacterial cells. Science doi: 10.1126/science.1222901. 10.1126/science.1222901 PMC3557511 22767897

[10] Basler M, Pilhofer M, Henderson GP, Jensen GJ, Mekalanos JJ (2012) Type VI secretion requires a dynamic contractile phage tail-like structure. Nature 483: 182–186. 22367545 10.1038/nature10846 PMC3527127

[11] Bönemann G, Pietrosiuk A, Diemand A, Zentgraf H, Mogk A (2009) Remodelling of VipA/VipB tubules by ClpV-mediated threading is crucial for type VI protein secretion. EMBO J 28: 315–325. 19131969 10.1038/emboj.2008.269 PMC2646146

[12] Pietrosiuk A, Lenherr ED, Falk S, Bönemann G, Kopp J, et al (2011) Molecular basis for the unique role of the AAA+ chaperone ClpV in type VI protein secretion. J Biol Chem 286: 30010–30021. 21733841 10.1074/jbc.M111.253377 PMC3191042

[13] Felisberto-Rodrigues C, Durand E, Aschtgen MS, Blangy S, Ortiz-Lombardia M, et al (2011) Towards a structural comprehension of bacterial type VI secretion systems: characterization of the TssJ-TssM complex of an *Escherichia coli* pathovar. PLoS Pathog 7: e1002386. 22102820 10.1371/journal.ppat.1002386 PMC3213119

[14] Durand E, Zoued A, Spinelli S, Watson PJ, Aschtgen MS, et al (2012) Structural characterization and oligomerization of the TssL protein, a component shared by bacterial type VI and type IVb secretion systems. J Biol Chem 287: 14157–14168. 22371492 10.1074/jbc.M111.338731 PMC3340138

[15] Ma LS, Narberhaus F, Lai EM (2012) IcmF family protein TssM exhibits ATPase activity and energizes type VI secretion. J Biol Chem 287: 15610–15621. 22393043 10.1074/jbc.M111.301630 PMC3346141

[16] Pukatzki S, Ma AT, Revel AT, Sturtevant D, Mekalanos JJ (2007) Type VI secretion system translocates a phage tail spike-like protein into target cells where it cross-links actin. Proc Natl Acad Sci U S A 104: 15508–15513. 17873062 10.1073/pnas.0706532104 PMC2000545

[17] Zheng J, Leung KY (2007) Dissection of a type VI secretion system in *Edwardsiella tarda* . Mol Microbiol 66: 1192–1206. 17986187 10.1111/j.1365-2958.2007.05993.x

[18] Hachani A, Lossi NS, Hamilton A, Jones C, Bleves S, et al (2011) Type VI secretion system in *Pseudomonas aeruginosa*: secretion and multimerization of VgrG proteins. J Biol Chem 286: 12317–12327. 21325275 10.1074/jbc.M110.193045 PMC3069435

[19] Ma AT, Mekalanos JJ (2010) In vivo actin cross-linking induced by *Vibrio cholerae* type VI secretion system is associated with intestinal inflammation. Proc Natl Acad Sci U S A 107: 4365–4370. 20150509 10.1073/pnas.0915156107 PMC2840160

[20] Suarez G, Sierra JC, Kirtley ML, Chopra AK (2010) Role of Hcp, a type 6 secretion system effector, of *Aeromonas hydrophila* in modulating activation of host immune cells. Microbiology 156: 3678–3688. 20798163 10.1099/mic.0.041277-0 PMC3068704

[21] Suarez G, Sierra JC, Erova TE, Sha J, Horneman AJ, et al (2010) A type VI secretion system effector protein, VgrG1, from *Aeromonas hydrophila* that induces host cell toxicity by ADP ribosylation of actin. J Bacteriol 192: 155–168. 19880608 10.1128/JB.01260-09 PMC2798274

[22] Miyata ST, Kitaoka M, Brooks TM, McAuley SB, Pukatzki S (2011) *Vibrio cholerae* requires the type VI secretion system virulence factor VasX to kill *Dictyostelium discoideum* . Infect Immun 79: 2941–2949. 21555399 10.1128/IAI.01266-10 PMC3191968

[23] Hood RD, Singh P, Hsu F, Güvener T, Carl MA, et al (2010) A type VI secretion system of *Pseudomonas aeruginosa* targets a toxin to bacteria. Cell Host Microbe 7: 25–37. 20114026 10.1016/j.chom.2009.12.007 PMC2831478

[24] Russell AB, Hood RD, Bui NK, LeRoux M, Vollmer W, et al (2011) Type VI secretion delivers bacteriolytic effectors to target cells. Nature 475: 343–347. 21776080 10.1038/nature10244 PMC3146020

[25] Li M, Le Trong I, Carl MA, Larson ET, Chou S, et al (2012) Structural basis for type VI secretion effector recognition by a cognate immunity protein. PLoS Pathog 8: e1002613. 22511866 10.1371/journal.ppat.1002613 PMC3325213

[26] Russell AB, Singh P, Brittnacher M, Bui NK, Hood RD, et al (2012) A widespread bacterial type VI secretion effector superfamily identified using a heuristic approach. Cell Host Microbe 11: 538–549. 22607806 10.1016/j.chom.2012.04.007 PMC3358704

[27] van Putten JPM, van Alphen LB, Wösten MM, de Zoete MR (2009) Molecular mechanisms of *Campylobacter* infection. Curr Top Microbiol Immunol 337: 197–229. 19812984 10.1007/978-3-642-01846-6_7

[28] Williams SG, Varcoe LT, Attridge SR, Manning PA (1996) *Vibrio cholerae* Hcp, a secreted protein coregulated with HlyA. Infect Immun 64: 283–289. 8557353 10.1128/iai.64.1.283-289.1996 PMC173757

[29] Ballister ER, Lai AH, Zuckermann RN, Cheng Y, Mougous JD (2008) *In vitro* self-assembly of tailorable nanotubes from a simple protein building block. Proc Natl Acad Sci U S A 105: 3733–3738. 18310321 10.1073/pnas.0712247105 PMC2268831

[30] Shalom G, Shaw JG, Thomas MS (2007) *In vivo* expression technology identifies a type VI secretion system locus in *Burkholderia pseudomallei* that is induced upon invasion of macrophages. Microbiology 153: 2689–2699. 17660433 10.1099/mic.0.2007/006585-0

[31] Aschtgen MS, Gavioli M, Dessen A, Lloubès R, Cascales E (2010) The SciZ protein anchors the enteroaggregative *Escherichia coli* Type VI secretion system to the cell wall. Mol Microbiol 75: 886–899. 20487285 10.1111/j.1365-2958.2009.07028.x

[32] Seydel A, Gounon P, Pugsley AP (1999) Testing the ‘+2 rule’ for lipoprotein sorting in the *Escherichia coli* cell envelope with a new genetic selection. Mol Microbiol 34: 810–821. 10564520 10.1046/j.1365-2958.1999.01647.x

[33] VanRheenen SM, Duménil G, Isberg RR (2004) IcmF and DotU are required for optimal effector translocation and trafficking of the *Legionella pneumophila* vacuole. Infect Immun 72: 5972–5982. 15385501 10.1128/IAI.72.10.5972-5982.2004 PMC517542

[34] Zusman T, Feldman M, Halperin E, Segal G (2004) Characterization of the *icmH* and *icmF* genes required for *Legionella pneumophila* intracellular growth, genes that are present in many bacteria associated with eukaryotic cells. Infect Immun 72: 3398–3409. 15155646 10.1128/IAI.72.6.3398-3409.2004 PMC415720

[35] Shrivastava S, Mande SS (2008) Identification and functional characterization of gene components of Type VI Secretion system in bacterial genomes. PLoS One 3: e2955. 18698408 10.1371/journal.pone.0002955 PMC2492809

[36] Bladergroen MR, Badelt K, Spaink HP (2003) Infection-blocking genes of a symbiotic *Rhizobium leguminosarum* strain that are involved in temperature-dependent protein secretion. Mol Plant Microbe Interact 16: 53–64. 12580282 10.1094/MPMI.2003.16.1.53

[37] Pukatzki S, McAuley SB, Miyata ST (2009) The type VI secretion system: translocation of effectors and effector-domains. Curr Opin Microbiol 12: 11–17. 19162533 10.1016/j.mib.2008.11.010

[38] Parker CT, Quiñones B, Miller WG, Horn ST, Mandrell RE (2006) Comparative genomic analysis of *Campylobacter jejuni* strains reveals diversity due to genomic elements similar to those present in *C. jejuni* strain RM1221. J Clin Microbio 44: 4125–4135. 10.1128/JCM.01231-06 PMC1698300 16943349

[39] Hill CW (1999) Large genomic sequence repetitions in bacteria: lessons from rRNA operons and Rhs elements. Res Microbiol 150: 665–674. 10673005 10.1016/s0923-2508(99)00125-4

[40] Skirrow MB, Jones DM, Sutcliffe E, Benjamin J (1993) Campylobacter bacteriaema in England and Wales, 1981–91. Epidemiol Infect 110: 567–573. 8519321 10.1017/s0950268800050986 PMC2272297

[41] Bernard CS, Brunet YR, Gavioli M, Lloubès R, Cascales E (2011) Regulation of type VI secretion gene clusters by sigma^54^ and cognate enhancer binding proteins. J Bacteriol 193: 2158–2167. 21378190 10.1128/JB.00029-11 PMC3133059

[42] van Alphen LB, Wuhrer M, Bleumink-Pluym NM, Hensbergen PJ, Deelder AM, et al (2008) A functional *Campylobacter jejuni maf4* gene results in novel glycoforms on flagellin and altered autoagglutination behaviour. Microbiology 154: 3385–3397.43. 18957592 10.1099/mic.0.2008/019919-0

[43] MacIntyre DL, Miyata ST, Kitaoka M, Pukatzki S (2010) The *Vibrio cholerae* type VI secretion system displays antimicrobial properties. Proc Natl Acad Sci U S A 107: 19520–19524. 20974937 10.1073/pnas.1012931107 PMC2984155

[44] Murdoch SL, Trunk K, English G, Fritsch MJ, Pourkarimi E, et al (2011) The opportunistic pathogen *Serratia marcescens* utilizes type VI secretion to target bacterial competitors. J Bacteriol 193: 6057–6069. 21890705 10.1128/JB.05671-11 PMC3194891

[45] Bouwman LI, Niewold P, van Putten JPM (2013) Basolateral invasion and trafficking of *Campylobacter jejuni* in polarized epithelial cells. PLoS ONE 8: e54759 doi: 10.1371/journal.pone.0054759. 23382959 10.1371/journal.pone.0054759 PMC3557275

[46] Chow J, Mazmanian SK (2010) A pathobiont of the microbiota balances host colonization and intestinal inflammation. Cell Host Microbe 7: 265–276. 20413095 10.1016/j.chom.2010.03.004 PMC2859213

[47] Jani AJ, Cotter PA Type VI secretion: not just for pathogenesis anymore. Cell Host Microbe 8: 2–6. 20638635 10.1016/j.chom.2010.06.012 PMC2913581

[48] Zheng J, Ho B, Mekalanos JJ (2011) Genetic analysis of anti-amoebae and anti-bacterial activities of the type VI secretion system in *Vibrio cholerae* . PLoS One 6: e23876. 21909372 10.1371/journal.pone.0023876 PMC3166118

[49] Bernard CS, Brunet YR, Gueguen E, Cascales E (2010) Nooks and crannies in type VI secretion regulation. J Bacteriol 192: 3850–3860. 20511495 10.1128/JB.00370-10 PMC2916374

[50] Mougous JD, Cuff ME, Raunser S, Shen A, Zhou M, et al (2006) A virulence locus of *Pseudomonas aeruginosa* encodes a protein secretion apparatus. Science 312: 1526–1530. 16763151 10.1126/science.1128393 PMC2800167

[51] Schell MA, Ulrich RL, Ribot WJ, Brueggemann EE, Hines HB, et al (2007) Type VI secretion is a major virulence determinant in *Burkholderia mallei* . Mol Microbiol 64: 1466–1485. 17555434 10.1111/j.1365-2958.2007.05734.x

[52] Grant KA, Belandia IU, Dekker N, Richardson PT, Park SF (1997) Molecular characterization of *pldA*, the structural gene for a phospholipase A from *Campylobacter coli*, and its contribution to cell-associated hemolysis. Infect Immun 65: 1172–1180. 9119448 10.1128/iai.65.4.1172-1180.1997 PMC175114

[53] Mota LJ, Journet L, Sorg I, Agrain C, Cornelis GR (2005) Bacterial injectisomes: needle length does matter. Science 307: 1278. 15731447 10.1126/science.1107679

[54] West NP, Sansonetti P, Mounier J, Exley RM, Parsot C, et al (2005) Optimization of virulence functions through glucosylation of *Shigella* LPS. Science 307: 1313–1317. 15731456 10.1126/science.1108472

[55] Corcionivoschi N, Clyne M, Lyons A, Elmi A, Gundogdu O, et al (2009) *Campylobacter jejuni* cocultured with epithelial cells reduces surface capsular polysaccharide expression. Infect Immun 77: 1959–1967. 19273563 10.1128/IAI.01239-08 PMC2681765

[56] Corcionivoschi N, Alvarez LA, Sharp TH, Strengert M, Alemka A, et al (2012) Mucosal reactive oxygen species decrease virulence by disrupting *Campylobacter jejuni* phosphotyrosine signaling. Cell Host Microbe 12: 47–59. 22817987 10.1016/j.chom.2012.05.018 PMC3749511

[57] Guerry P, Szymanski CM (2008) *Campylobacter* sugars sticking out. Trends Microbiol 16: 428–435. 18707886 10.1016/j.tim.2008.07.002

[58] Lertpiriyapong K, Gamazon ER, Feng Y, Park DS, Pang J, et al (2012) *Campylobacter jejuni* type VI secretion system: roles in adaptation to deoxycholic acid, host cell adherence, invasion, and *in vivo* colonization. PLoS ONE 7: e42842. 22952616 10.1371/journal.pone.0042842 PMC3428339

[59] van Mourik A, Bleumink-Pluym NM, van Dijk L, van Putten JP, Wösten MM (2008) Functional analysis of a *Campylobacter jejuni* alkaline phosphatase secreted via the Tat export machinery. Microbiology 154: 584–592. 18227262 10.1099/mic.0.2007/012120-0

[60] van Alphen LB, Bleumink-Pluym NM, Rochat KD, van Balkom BW, Wösten MM, et al (2008) Active migration into the subcellular space precedes *Campylobacter jejuni* invasion of epithelial cells. Cell Microbiol 10: 53–66. 18052944 10.1111/j.1462-5822.2007.01014.x

