# Supplementary figures and images for: Identification of a Functional Type VI Secretion System in Campylobacter jejuni Conferring Capsule Polysaccharide Sensitive Cytotoxicity
Source: PLoS Pathog. 2013 May 30;9(5):e1003393. doi: 10.1371/journal.ppat.1003393 (PMC3667781; doi:10.1371/journal.ppat.1003393)

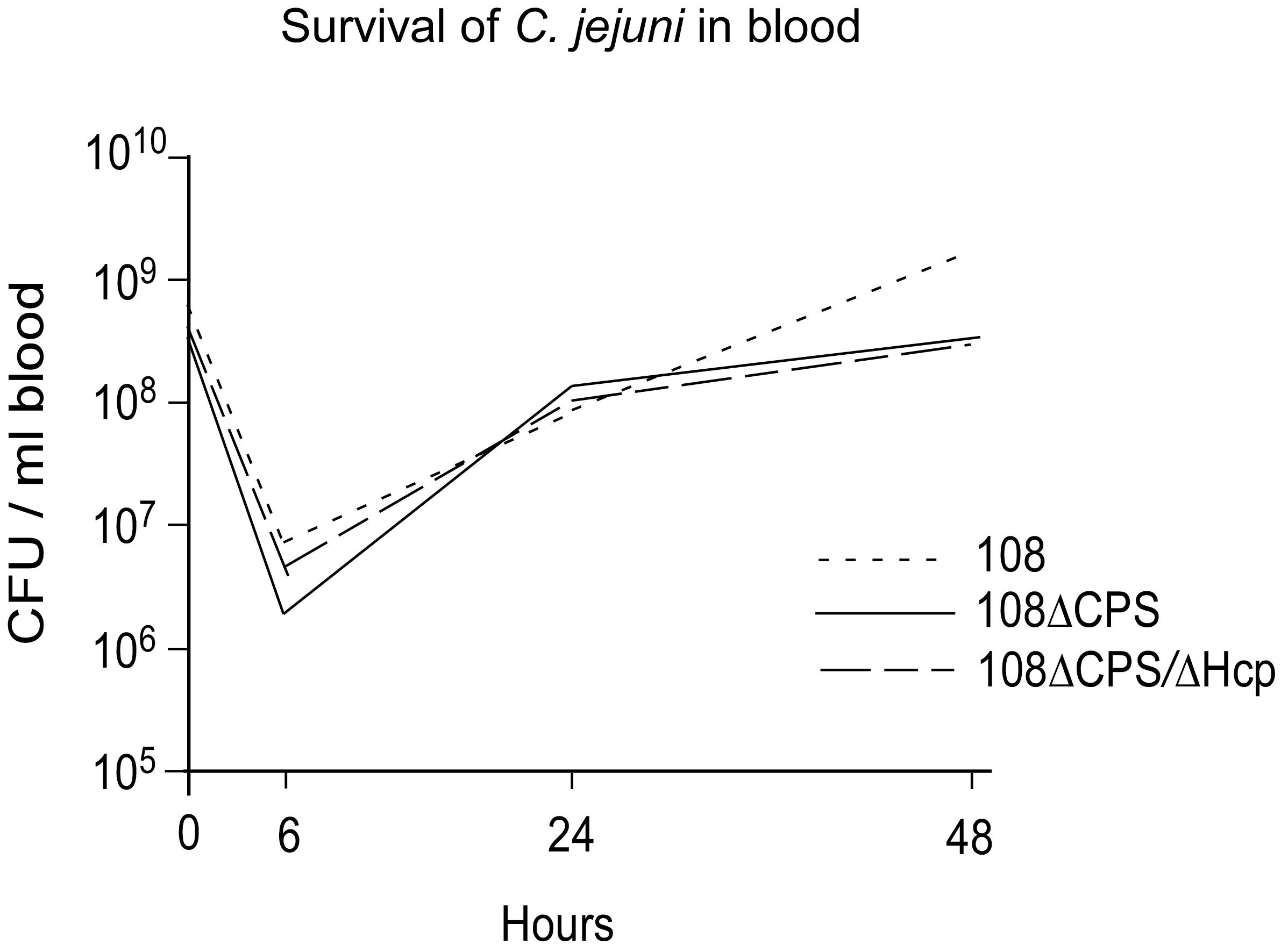

Supplement: Figure S1 — Growth of C. jejuni strain 108, 108ΔCPS and 108ΔCPS/ΔHcp in blood. (TIF) [file ppat.1003393.s001.tif]
