# Supplementary material for: Identification of a Functional Type VI Secretion System in Campylobacter jejuni Conferring Capsule Polysaccharide Sensitive Cytotoxicity
Source: PLoS Pathog. 2013 May 30;9(5):e1003393. doi: 10.1371/journal.ppat.1003393 (PMC3667781; doi:10.1371/journal.ppat.1003393)
Supplement: Table S1 — Strains and plasmids used in this study. (DOC) [file ppat.1003393.s002.doc]

**Table S1.** Strains and plasmids used in this study.

| **Strain/plasmid** | **Relevant characteristics** | **Source / Reference** |
| --- | --- | --- |
|
| ***C. jejuni*** |  |  |
| 108 wild type (129108) | Bacteremia (intestinal isolate) | [1] |
| 108ΔHcp | 108 *hcp::Cm* | This study |
| 108ΔTssM | 108 *tssM::Cm* | This study |
| 108ΔCPS | 108 *kpsM::Cm* | This study |
| 108ΔCPS*/*ΔHcp | 108 *kpsM::Cm hcp::Km* | This study |
| 108ΔCPS/ΔTssM | 108 *kpsM::Cm tssM::Km* | This study |
| 108ΔRpoN | 108 *rpoN::Cm* | [2] |
| NCTC81116 | Human enteritis | [3] |
| NCTC81116ΔCPS | 81116 *kpsM::tet* | This study |
|  |  |  |
| ***CJIE3-positive strains*** |  |  |
| 108; 202606; 205223; 209223 | Bacteremia | [1] |
| NCTC 12502 (LCDC C2603) | Human isolate | [4] |
| RM1221 | Chicken intestine | [5] |
| C01965; C019168;  C356 | Human intestine  Chicken intestine | [6,7] |
| 117;  C10; C626; C631 | Human intestine  Swine intestine | This study |
|  |  |  |
| ***CJIE3-negative strains*** |  |  |
| 81176;  A3004 | Human enteritis  Human enteritis | [8]  [9] |
| 07479; 41239B; 40707L  127955850312  5003  ATCC49301; ATCC33291; | Human enteritis  Human enteritis  Cattle isolate  Human enteritis | [10]  [10]  D. Grove, Liverpool  ATCC |
| C011300; C011338; C011672; C017289; C013500; C012446;  C012599; C013199 | Unknown | J.A. Frost, UK, [6] |
| D3141; D3226; D3468;  ATCC 43446 (CCUG 10950) | Human enteritis | [11] |
| BAA527 (HB95-29);  BAA529 (INP59); | GBS  GBS | [11] |
| 233.95; 260.94; 308.95; GB1; GB5; GB11; GB18; GB23; GB26; GB27 | all GBS | [12] |
| E98623; 386.96; 21.97 | Human enteritis | [12] |
| 11271; 11279 | Unknown | [13] |
| 480 | Human enteritis | [14] |
| 100756; 105713; 132960; 146719 | Human enteritis | H. Endtz, Rotterdam |
| 201191; 205224; 206470; 206710; 207251; 207252; 209071; 209755 | Bacteremia | H. Endtz, Rotterdam |
| 210388 | Unknown | H. Endtz, Rotterdam |
| NCTC11168 (5636/77 Lucitt) | Human enteritis | [15] |
| C9; C12; C608; C621; C627 | Human enteritis | [16] |
| C618 | Chicken isolate | [16] |
|  |  |  |
| ***CJIE3-positive C. coli strains*** | |  |
| Han 35; Han 153 | Chicken isolate | [17] |
|  |  |  |
| ***CJIE3-negative C. coli strains*** | |  |
| 2371; Han36;  K1102/03 | Chicken stool  Chicken liver | [17] |
| UA417 (LCDC C2633) | Human stool | [18] |
| H1 | Chicken stool | [19] |
|  |  |  |
| ***E. coli*** |  |  |
| PC2955 | *relA1,* Φ*80dlacZ* ΔM15, *phoA8, hsdR17, recA1 endA1, gyrA96, thi-1, luxS, glnV44* | Netherlands Culture Collection of Bacteria |
| CYS21 | F^-^, Cm^R^, *mcr*A, *end*A1, Δ(*mrr-hsd*RMS*-mcr*BC), Φ*80dlacZ*ΔM15, Δ*lac*X74, *rec*A1, Δ*(ara,leu)7697, ara*D139, *galU, galK, nupG, rps*I, *ccdB^+^.* | Delphi Genetics SA |
| SE1 | F^-^, Cm^R^*, ompT, lon, hsdS_B_, gal, dcm,* DE3, *ccdB^+^* | Delphi Genetics SA |
|  |  |  |
| **Plasmids** |  |  |
| pGEM-T Easy | PCR cloning vector, Amp^R^ | Promega |
| pSCodon1 | Staby ^TM^ Codon T7 expression kit encoding tRNA genes of the six rare codons | Delphi Genetics SA |
| pJET1.1/blunt | Cloning vector, Amp^R^ | Fermentas GMBH |
| pAV35 | pBluescript KS M13^+^ ::Cm^R^ (*C. coli*) | [20] |
| pMW2 | pBluescript KS M13^+^ ::Km^R^ (pILL550) | [2] |
| pMA1 | Conjugative expression vector,Cm^R^ | [21] |
| pHcp | Expression plasmid pMA1 containing *hcp* | This study |

**References**

1. Endtz HP, Giesendorf BA, van Belkum A, Lauwers SJ, Jansen WH, et al. (1993) PCR-mediated DNA typing of *Campylobacter jejuni* isolated from patients with recurrent infections. Res Microbiol 144: 703-708.

2. Wösten, M (1997) Initiation of transcription and gene organization in *Campylobacter jejuni*. PhD thesis, Utrecht University.

3. Palmer SR, Gully PR, White JM, Pearson AD, Suckling WG, et al. (1983) Water-borne outbreak of Campylobacter gastroenteritis. Lancet 1: 287-290.

4. Owen RJ, Fitzgerald C, Sutherland K, Borman P (1994) Flagellin gene polymorphism analysis of *Campylobacter jejuni* infecting man and other hosts and comparison with biotyping and somatic antigen serotyping. Epidemiol Infect 113:221-234.

5. Fouts DE, Mongodin EF, Mandrell RE, Miller WG, Rasko DA, et al. (2005) Major structural differences and novel potential virulence mechanisms from the genomes of multiple *Campylobacter* species. PLoS Biol 3: e15.

6. Gaasbeek EJ, Wagenaar JA, Guilhabert MR, Wösten MM, van Putten JP, et al. (2009) A DNase encoded by integrated element CJIE1 inhibits natural transformation of *Campylobacter jejuni*. J Bacteriol 191: 2296-2306.

7. Gaasbeek EJ, Wagenaar JA, Guilhabert MR, van Putten JP, Parker CT, et al. (2010) Nucleases encoded by the integrated elements CJIE2 and CJIE4 inhibit natural transformation of *Campylobacter jejuni*. J Bacteriol 192: 936-941.

8. Black RE, Levine MM, Clements ML, Hughes TP, Blaser MJ (1988) Experimental *Campylobacter jejuni* infection in humans. J Infect Dis 157: 472-479.

9. Committee on Public Health Risk Assessment of Poultry Inspection Programs, Food and Nutrition Board, National Research Council (1987) Poultry Inspection: The Basis for a Risk-Assessment Approach. The National Academies Press.

10. Harrington CS, Thomson-Carter FM, Carter PE (1999) Molecular epidemiological investigation of an outbreak of *Campylobacter jejuni* identifies a dominant clonal line within Scottish serotype HS55 populations. Epidemiol Infect 122: 367-375.

11. Nachamkin I, Engberg J, Gutacker M, Meinersman RJ, Li CY, et al. (2001) Molecular population genetic analysis of *Campylobacter jejuni* HS:19 associated with Guillain-Barre syndrome and gastroenteritis. J Infect Dis 184: 221-226.

12. Godschalk PC, Bergman MP, Gorkink RF, Simons G, van den Braak N, Lastovica AJ, Endtz HP, Verbrugh HA, van Belkum A (2006) Identification of DNA sequence variation in *Campylobacter jejuni* strains associated with the Guillain-Barré syndrome by high-throughput AFLP analysis. BMC Microbiol 6: 32.

13. Nuijten PJ, van Asten FJ, Gaastra W, van der Zeijst BA (1990) Structural and functional analysis of two *Campylobacter jejuni* flagellin genes. J Biol Chem 265: 17798-17804.

14. King V, Wassenaar T, Van der Zeijst BAM, Newell DG (1991) Variations in C*ampylobacter jejuni* flagellin, and flagellin genes, during *in vivo* and *in vitro* passage. Micr. Ecol. Health Dis. 4: 135-140.

15. Skirrow MB (1977) Campylobacter enteritis: a "new" disease. Br Med J2: 9-11.

16. Dingle KE, Colles FM, Ure R, Wagenaar JA, Duim B, Bolton FJ, Fox AJ, Wareing DR, Maiden MC (2002) Molecular characterization of *Campylobacter jejuni* clones: a basis for epidemiologic investigation. Emerg Infect Dis 8: 949-955.

17. Krause-Gruszczynska M, van Alphen LB, Oyarzabal OA, Alter T, Hänel I, et al. (2007) Expression patterns and role of the CadF protein in *Campylobacter jejuni* and *Campylobacter coli*. FEMS Microbiol Lett 274: 9-16.

18. Wenman WM, Chai J, Louie TJ, Goudreau C, Lior H, Newell DG, Pearson AD, Taylor DE (1985) Antigenic analysis of *Campylobacter* flagellar protein and other proteins. J Clin Microbiol 21: 108–112.

19. de Zoete MR, Keestra AM, Roszczenko P, van Putten JP. (2010) Activation of human and chicken Toll-like receptors by *Campylobacter* spp. Infect Immun78(3): 1229-1238.

20. van Vliet AH, Wooldridge KG, Ketley JM (1998) Iron-responsive gene regulation in a *Campylobacter jejuni fur* mutant. J Bacteriol 180: 5291-5298.

21. van Mourik A, Bleumink-Pluym NM, van Dijk L, van Putten JP, Wösten MM (2008) Functional analysis of a *Campylobacter jejuni* alkaline phosphatase secreted via the Tat export machinery. Microbiology 154: 584-592.
