# Supplementary material for: Identification of a Functional Type VI Secretion System in Campylobacter jejuni Conferring Capsule Polysaccharide Sensitive Cytotoxicity
Source: PLoS Pathog. 2013 May 30;9(5):e1003393. doi: 10.1371/journal.ppat.1003393 (PMC3667781; doi:10.1371/journal.ppat.1003393)
Supplement: Table S2 — Primers used for cloning and sequencing of the T6SS locus of C. jejuni strain 108. (DOC) [file ppat.1003393.s003.doc]

**Table S2:** Primers used for cloning and sequencing of the T6SS locus of *C. jejuni* strain 108.

| **Primer Number** | **Primer Name** | **Primer sequence (5’to 3’)** |
| --- | --- | --- |
|  |  |  |
| 189 | Hcp fwd | CTACAAAATTGGAAGATGCAATTAT |
| 190 | Hcp rev | TAAGCTTTGCCCTCTCTCCA |
| 221 | tagH | CACTCAAAATCTTTAATATACTTTTCC |
| 222 | tssF-1 | GATCCTGATATAGAAAGAATTATTGAA |
| 223 | tssF-2 | CTCACAAAAGGAGTTAATACTAGC |
| 228 | tssA-1 | GCTCAAGTTGCACCACAACTTC |
| 229 | tssA-2 | AAGAGTTGTACACCCTCTATCC |
| 245 | tssM-1 | TCTAGAAGGGGTGGATGAGC |
| 246 | tssM-2 | TTGCAGCAAAACCTAAATCCTC |
| 258 | CJE 1138 | TTCTTACAGGGCAACATTGG |
| 275 | CJE 1150 | TCCGCATGCATTTAATCTTC |
| 278 | VgrG-1 | TTGATTTAAAGACTAAGCCTTCTC |
| 279 | VgrG-2 | AACCTTCTTCATTAACACCTATGG |
