# Supplementary material for: Identification of a Functional Type VI Secretion System in Campylobacter jejuni Conferring Capsule Polysaccharide Sensitive Cytotoxicity
Source: PLoS Pathog. 2013 May 30;9(5):e1003393. doi: 10.1371/journal.ppat.1003393 (PMC3667781; doi:10.1371/journal.ppat.1003393)
Supplement: Table S3 — Primers used for construction of mutants and expression vectors. (DOC) [file ppat.1003393.s004.doc]

**Table S3.** Primers used for construction of mutants and expression vectors.

| **Primer** | **Primer sequence (5’to 3’)** |
| --- | --- |
|  |  |
| Hcp fwd | CTACAAAATTGGAAGATGCAATTAT |
| Hcp rev | TAAGCTTTGCCCTCTCTCCA |
| del Hcp BamHI fwd | CGGGATCCAGTCGAAGTTCATTGGTTTAG |
| del Hcp BamHI rev | CGGGATCCTCATCTTCATGACCCGATTTA |
| tssM forw | TCTAGAAGGGGTGGATGAGC |
| tssM rev | AAAGCCGCAATTGTAAAAGATCC |
| stabyHCPfwd | CCTCTAGACTTTAAGAAGGAGATATACAT**ATG**GCTGAACCAGCGTTTATA |
| stabyHCPrev | GTGCTCGAGGCCCTGAAAATACAGGTTTTCAGCTTTGCCCTCTCTCCAATC |
| HCP Complementation fwd | CCGCTCGAGAAGGAGTTTTTTT**ATG**GCTGAACCAGCG |
| HCP Complementation rev | CTAGTCTAGATTAAGCTTTGCCCTCTCTCC |
| CJIE3 fwd | GTATCATTTGTTGCTTTGGC |
| CJIE3 rev | TTGAGAGCATTAACTAGC |
| KpsM fwd | CAGCTATTCCTTCATCTACATCA |
| KpsM rev | AGCTTTTATCTTGGGCTATG |
| KpsM BglII fwd | ATTAGATCTCCTACTTGGCTAACTCTT |
| KpsM BglII rev | AAGAGAGATCTATTTGCCTGAGTTCC |

Underlined sequences represent restriction sites used in cloning of the PCR products.
